# Prevalence and clinical relevance of negative histology after transsphenoidal surgery for pituitary neuroendocrine tumors

**DOI:** 10.3389/fendo.2026.1933467

**Published:** 2026-09-15

**Authors:** Gilles De Cannière, Christian Raftopoulos, Dominique Maiter, Loic de Nijs, Lina Daoud, Stefan Matei Constantinescu, Orsalia Alexopoulou, Edward Fomekong

**Affiliations:** 1 Faculty of Medicine, Université Catholique de Louvain, Brussels, Belgium; 2 Department of Neurosurgery, Saint-Luc University Hospital, Brussels, Belgium; 3 Department of Neurosurgery, Clinique Notre-Dame de Grace (CNDG), Gosselies, Belgium; 4 Department of Pathology, Saint-Luc University Hospital, Brussels, Belgium; 5 Department of Endocrinology and Nutrition, Saint-Luc University Hospital, Brussels, Belgium

**Keywords:** acromegaly, Cushing’s disease, microadenoma, negative histology, pituitary neuroendocrine tumor, transsphenoidal surgery, tumor mislocalization

## Abstract

**Purpose:**

Negative histology (NH), defined as normal pituitary tissue without histopathological evidence of tumor despite clinical, biochemical and radiological or functional evidence of a pituitary neuroendocrine tumor (PitNET), is well described in Cushing’s disease but poorly characterized in other subtypes. We assessed its prevalence, mechanisms, and clinical implications.

**Methods:**

We retrospectively analyzed 651 patients who underwent transsphenoidal surgery for a PitNET (1996–2023). Epidemiological analyses included the entire cohort; clinical and prognostic analyses were restricted to microadenomas of the three main functional subtypes (ACTH, PRL, GH).

**Results:**

NH occurred in 28.1% of ACTH-, 28.1% of PRL-, and 11.4% of GH-secreting tumors, and was strongly associated with microadenomas. Baseline and hormonal characteristics were similar between NH and PH cases, except for lower IGF-1 levels in GH NH tumors. At 6 weeks, remission rates were lower in NH for ACTH (60% vs. 93.2%, *p* < 0.01) and PRL (50% vs. 79.3%, *p* = 0.03), but similar in GH. In ACTH tumors, mislocalization accounted for 66.7% of unfavorable NH outcomes and may explain the excess of failures versus PH cases. Mislocalization was rare (6.7%) in PRL tumors and not reported in GH tumors.

**Conclusion:**

NH is heterogeneous, arising from three mechanisms whose contributions vary by subtype. In ACTH tumors, mislocalization predominates and is associated with lower early remission. In PRL tumors, mislocalization is uncommon but may contribute to lower early remission. In GH tumors, NH likely reflects technical or histological limitations and carries a favorable prognosis.

## Introduction

Pituitary neuroendocrine tumors (PitNETs), previously known as pituitary adenomas, are common intracranial neoplasms arising from the adenohypophysis. They can present with a wide spectrum of clinical manifestations, ranging from incidental nonfunctioning tumors to highly morbid hormone-secreting lesions. Their clinical impact depends on both mass effects and hormonal hypersecretion, necessitating a multidisciplinary approach involving neurosurgeons, ophthalmologists, radiologists, and endocrinologists (1, 2). Although advances in imaging and endocrinological testing have improved the diagnostic accuracy and outcomes of PitNETs, histopathological confirmation remains the gold standard for definitive diagnosis and subtype classification (3).

Negative histology (NH) refers to the absence of histological confirmation despite compelling clinical or biochemical and radiological or functional evidence of a PitNET. It has been best documented in corticotroph tumors associated with Cushing’s disease, in which reported rates range widely from 5% to 44% (see **Supplementary Table 1** for a narrative literature review). Outside this subtype, NH is only occasionally reported, mostly in prolactin (PRL)-secreting tumors with a prevalence ranging from 3% to 37.5% (4–6), and in somatotroph tumors with a prevalence of 3% to 7% (6, 7).

Despite these observations, the true prevalence and underlying causes of NH remain incompletely characterized. Three main hypotheses may explain the occurrence of NH (8, 9). First, tumor tissue may be inadvertently replaced by normal pituitary tissue during the sampling. Second, limitations in histopathological analysis, such as small lesion size or atypical tumor features, may hinder accurate identification of tumor tissue. The existence of hormone-secreting pituitary hyperplasia has also been suggested but lacks a formal definition (10, 11). These first two hypotheses could be seen as “logistical failures” and should be associated with a good prognosis as they are not linked to the surgery itself. Finally, the third hypothesis is that incorrect tumor localization may result in resection of normal pituitary tissue instead of the actual lesion. In this situation, NH should be associated with a lower prognosis as the surgery missed the actual lesion, but some authors suggested that remission in such cases may still occur and could be explained by necrosis of the PitNET due to accidental vascular injury during the surgery (12).

From a clinical perspective, the prognostic significance of NH remains controversial. In ACTH-secreting tumors, a recent meta-analysis found that NH findings carry a negative prognostic value (13), though several case series not included in that analysis have called this conclusion into question (14–19).

The aim of this study was to investigate the prevalence, causes and clinical significance of NH across PitNET subtypes and to evaluate its implications for postoperative management and surgical decision-making in our center.

## Materials and methods

### Study population

We retrospectively reviewed the clinical files of all patients who underwent a first transsphenoidal surgery for a suspected PitNET at Saint-Luc Hospital in Brussels, Belgium, between January 1, 1996, and December 31, 2023. The exclusion criteria included the absence of postoperative endocrinology follow-up at 6 weeks, missing or uninterpretable histopathological material, no preoperative or postoperative MRI results, the absence of a visible tumor on preoperative imaging (with the exception of Cushing’s disease cases for which inferior petrosal sinus sampling [IPSS] was in favor of a pituitary origin) and the presence of an alternative diagnosis on pathology (e.g., lymphocytic hypophysitis or gangliocytoma).

The study was conducted in two sequential analytical steps. First, the entire cohort was analyzed to determine the prevalence of negative histology (NH) across PitNET subtypes and according to tumor size.

Detailed prognostic and hormonal analyses were subsequently focused on the three most common functional subtypes, namely adrenocorticotropic hormone (ACTH)-, prolactin (PRL)-, and growth hormone (GH)-secreting tumors, because these groups provided sufficient sample size and clinically relevant outcome assessment. Other subtypes were included only in the descriptive analysis of the overall cohort.

Because tumor size is a major determinant of surgical outcome, multivariable logistic regression analyses adjusting for maximum tumor diameter were first performed in the overall cohort of ACTH-, PRL-, and GH-secreting tumors. Secondary analyses restricted to microadenomas were then conducted to reduce residual heterogeneity related to tumor size.

### Clinical and biochemical evaluation

The preoperative assessment included a comprehensive endocrinological evaluation of baseline hormone levels and dynamic testing as appropriate in ACTH- and GH-secreting tumors. Clinical presentation (distinguishing symptoms related to hormone hypersecretion from mass effect) and duration of symptoms were extracted from the medical records, with symptom duration defined as the interval between the first patient-reported symptoms and the date of surgery. Postoperative hormonal evaluation was conducted at 5 days and 6 weeks, with further follow-up visits guided by tumor subtype and clinical evolution.

### Radiological assessment

All patients underwent preoperative MRI of the sellar region, which was assessed by experienced neuroradiologists. In patients with Cushing’s disease and negative or inconclusive MRI findings, the diagnosis and surgical indication had to be supported by IPSS. In some cases, 11C-methionine PET imaging was used as an adjunct technique. These cases were considered as having functional evidence of a pituitary source despite the absence of a visible lesion on MRI.

Confirmed tumor mislocalization was defined as cases in which the initial surgical exploration did not target the true tumor location, as subsequently demonstrated histologically by the identification of a functioning tumor at a different site after repeat surgery. Cases where follow-up imaging showed a tumor at another localization without anatomopathological confirmation were categorized as suspected tumor mislocalization. Cases with persistent or recurrent tumors at the initial surgical site were therefore not considered mislocalized.

### Surgical technique

All operations were performed using a microscopic endonasal transsphenoidal approach with neuronavigation assistance. Intraoperative findings, including visualization of adenomatous tissue, evidence of hemorrhage, and technical limitations in specimen retrieval, were systematically documented. At least one of two senior pituitary surgeons (C.R., E.F.) was present at every procedure, either as primary surgeon or assistant, ensuring consistency in surgical technique across the cohort.

### Histopathological analysis

All histopathological analyses were performed by experienced pituitary pathologists at our tertiary referral center. Tissue samples were formalin-fixed and processed using standard paraffin-embedding techniques. Hematoxylin and eosin staining was performed in all cases, as well as reticulin staining to assess the integrity of the acinar framework and immunohistochemistry to assess pituitary hormones content. Immunostaining for pituitary transcription factors was implemented only in recent years and in a small subset of cases; consequently, it was not incorporated into the PitNET classification in this study. Histopathological confirmation was defined as unequivocal adenomatous morphology, including disruption of the reticulin network, with or without immunohistochemical evidence of hormone production. NH was defined as the presence of normal pituitary tissue without histopathological evidence of adenoma despite compatible clinical or biochemical, and radiological or functional findings. Focal pituitary hyperplasia was neither specifically investigated nor routinely documented in the original pathology reports and was thus not evaluated in the present study.

PitNETs were classified according to their preoperative functional diagnosis based on clinical presentation and hormonal evaluation, rather than on histopathological findings. Accordingly, cases in which histology suggested a different functional subtype than initially suspected (e.g., a pathology-confirmed GH-containing tumor in a patient with a clinical and biochemical preoperative diagnosis of prolactinoma) were analyzed in the group corresponding to the preoperative functional diagnosis.

### Outcome definitions

Remission criteria were defined according to postoperative biochemical normalization of hormone secretion at 6 weeks and the last follow-up. For Cushing’s disease, remission was defined as postoperative morning serum cortisol levels below 50 nmol/L. For PRL-secreting tumors, remission was defined as normalization of serum prolactin levels according to sex-specific laboratory reference range. For GH-secreting tumors, remission was defined as normalization of age-adjusted insulin-like growth factor 1 (IGF-1).

An unfavorable outcome was defined as the absence of postoperative biochemical remission or disease recurrence during follow-up. Accordingly, any patient requiring additional postoperative treatment, whether medical or surgical, was classified as having an unfavorable outcome.

### Statistical analysis

Group comparisons used Fisher’s exact test for categorical variables and the Mann–Whitney U test for continuous variables. For comparisons of continuous variables between more than two groups with unequal variances, Welch’s one-way ANOVA followed by appropriate *post-hoc* testing was applied. Effect sizes were estimated using Hedges’ g for continuous outcomes and odds ratios (OR) for binary outcomes. *p* < 0.05 was considered statistically significant. Statistical analyses were performed using R and graphical representations were obtained using Matplotlib.

Multivariable logistic regression analyses were performed with remission status as dependent variable and histological status and maximum tumor diameter as covariates. Additional exploratory models including age, sex, and BMI were also evaluated.

This study was reported in accordance with the STROBE guidelines.

## Results

The study included 651 patients (female-to-male ratio of 1.3) who underwent a first transsphenoidal surgery for a PitNET. The mean age at surgery was 48.5 years (range 8–87). The baseline characteristics of the cohort are summarized in **Table 1**. Based on preoperative diagnosis, the most frequent subtype of PitNET was non-functioning tumors (47%) followed by PRL- (17%), GH- (16%) and ACTH-secreting tumors (15%). Most operated cases were macroadenomas (75.3%). Six Cushing’s disease cases without a visible tumor on the preoperative MRI were included as radiologically occult lesions, as they were all (6/6) confirmed by IPSS. In one case, the tumor was also confirmed by 11C-methionine PET, while in another case, PET was inconclusive.

**Table 1:** Baseline characteristics of patients undergoing a first surgery for PitNET.

Patient and tumor characteristics	Overall cohort (n = 651)
Demographics	
Age at surgery, years — mean (SD)	48.5 (16.4)
Female sex, n (%)	367 (56.4)
BMI, kg/m² — mean (SD)	28.1 (6.1)
Tumor characteristics	
Suspected preoperative subtype, n (%)	
PRL	114 (17.5)
GH	105 (16.1)
ACTH	96 (14.7)
TSH	11 (1.7)
LH/FSH	8 (1.2)
Plurihormonal	14 (2.2)
Non-functioning	303 (46.5)
Tumor size on MRI, n (%)	
Macroadenoma	490 (75.3)
Microadenoma	155 (23.8)
Radiologically occult lesion	6 (0.9)
Histology, n (%)	
Positive histology	569 (87.4)
Negative histology	82 (12.6)

ACTH, adrenocorticotropic hormone; PRL, prolactin; GH, growth hormone; TSH, thyroid-stimulating hormone; LH/FSH, luteinizing hormone/follicle-stimulating hormone; NF, non-functioning. Demographic, clinical, radiological, and histopathological characteristics of the overall cohort.

As described in Methods, analyses were performed in two steps. First, the entire cohort was analyzed to determine the prevalence of NH according to subtype and tumor size, as well as for the multivariable logistic regression analyses in the three most common functional subtypes (ACTH, PRL, GH). Second, detailed clinical and prognostic analyses were restricted to microadenomas in these subtypes.

### Prevalence of NH

The overall prevalence of NH was 12.6%, differing by preoperative functional diagnosis: 28.1% (27/96) for ACTH-secreting tumors, 28.1% (32/114) for PRL-secreting tumors, 11.4% (12/105) for GH-secreting tumors, 7.1% (1/14) for plurihormonal-secreting tumors and 3.3% (10/303) for non-functioning tumors.

Overall, 78% (64/82) of NH cases were microadenomas or radiologically occult lesions and a strong, statistically significant, positive association was observed between microadenoma prevalence and the prevalence of NH across PitNET subtypes (r = 0.87, R² = 0.77, p < 0.01; **Figure 1A**).

**Figure 1:**
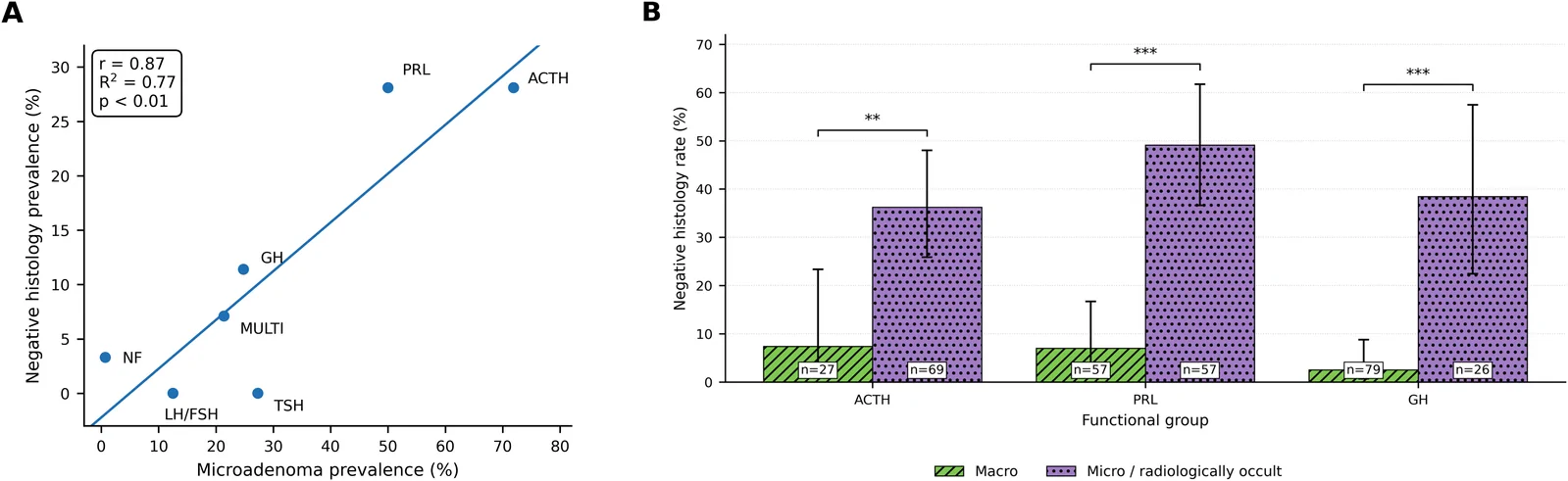
Prevalence of negative histology across PitNET subtypes. **(A)** Association between microadenoma prevalence and negative histology prevalence across PitNET subtypes. **(B)** Proportion of tumors with NH according to functional subtype and tumor size (microadenomas, macroadenomas, and radiologically occult lesions). Only ACTH-, PRL-, and GH-secreting tumors are shown, as NH cases were too infrequent in other subtypes to allow meaningful visualization or comparison. ACTH, adrenocorticotropic hormone; PRL, prolactin; GH, growth hormone; PLURI, plurihormonal; TSH, thyroid-stimulating hormone; LH/FSH, luteinizing hormone/follicle-stimulating hormone; NF, non-functioning; PitNET, pituitary neuroendocrine tumor.

When tumors were stratified by size, NH was significantly more frequent in microadenomas than in macroadenomas across the three most common functional subtypes (**Figure 1B**). In ACTH-secreting tumors, NH occurred in 36.2% of microadenomas compared with 7.4% of macroadenomas (*p* < 0.01). Similarly, in PRL-secreting tumors, NH was observed in 49.1% *vs.* 7.0%, respectively (*p* < 0.001), and in GH-secreting tumors, in 38.5% of microadenomas *vs.* 2.5% of macroadenomas (*p* < 0.001).

### Baseline characteristics of NH and positive histology cases

When comparing the NH and positive histology (PH) groups across microadenomas in the three most common functional subtypes (ACTH, n = 69; PRL, n = 57; GH, n = 26), baseline demographic characteristics were not statistically different (**Table 2**).

**Table 2:** Comparison of baseline characteristics between NH and PH cases in patients with microadenomas, across the three most common functioning subgroups.

Functional subgroup	Positive Histology (n)	Negative Histology (n)	p
ACTH	44	25	
**Demographics**			
Age at surgery, years — mean (SD)	42.3 (14)	41.7 (12.0)	0.89
Female sex, n (%)	35 (79.5)	21 (84.0)	0.76
BMI, kg/m² — mean (SD)	31.8 (8.2)	29.1 (6.6)	0.23
**Endocrine symptoms**			
Prevalence, n (%)	41 (93.2)	22 (88.0)	0.66
Duration of symptoms, years — mean (SD)	3 (3.2)	1.8 (1.3)	0.23
Incidentaloma, n (%)	5 (11.4)	4 (16.0)	0.71
Maximal diameter*, mm — median (IQR)	6.0 (4.8–7.0)	4.1 (3.0–5.3)	< 0.01
Inferior petrosal sinus sampling — n, (%)	16 (36.4)	14 (56.0)	0.14
Lateralized — n, (%)	15 (93.8)	11 (78.6)	0.45
11C-methionine PET — n, (%)	2 (4.5)	3 (12.0)	0.34
Positive — n, (%)	1 (50.0)	2 (66.7)	0.30
Median follow-up duration, years — median (IQR)	5.0 (2.5–12.1)	3.0 (2.0–9.5)	0.15
PRL	29	28	
**Demographics**			
Age at surgery, years — mean (SD)	30.8 (11.2)	33.4 (9.5)	0.19
Female sex, n (%)	28 (96.6)	24 (85.7)	0.19
BMI, kg/m² — mean (SD)	23.8 (4.6)	25.2 (5.5)	0.35
**Endocrine symptoms**			
Prevalence, n (%)	27 (93.1)	26 (92.9)	1.00
Duration of symptoms, years — mean (SD)	5.0 (4.6)	6.5 (5.0)	0.28
Incidentaloma, n (%)	0 (0.0)	1 (3.6)	0.49
Maximal diameter, mm — median (IQR)	7.0 (6.0–8.5)	5.8 (4.9–7.0)	0.01
Median follow-up duration, years — median (IQR)	5.7 (1.1–10.9)	2.9 (0.7–7.5)	0.20
GH	16	10	
**Demographics**			
Age at surgery, years — mean (SD)	47.8 (13.1)	46.9 (17.3)	0.98
Female sex, n (%)	7 (43.8)	6 (60.0)	0.69
BMI, kg/m² — mean (SD)	29.2 (4.4)	27.2 (6.1)	0.42
**Endocrine symptoms**			
Prevalence, n (%)	16 (100.0)	10 (100)	1.00
Duration of symptoms, years — mean (SD)	8.4 (12.8)	2.8 (1.2)	0.97
Incidentaloma, n (%)	1 (6.2)	0 (0.0)	1.00
Maximal diameter, mm — median (IQR)	7.2 (6.0–8.2)	5.5 (4.2–6.0)	0.02
Median follow-up duration, years — median (IQR)	3.8 (1.9–7.2)	2.5 (1.6–5.5)	0.58

Clinical, radiological, and biological characteristics are compared between NH and PH groups within ACTH-, PRL-, and GH-secreting tumors.

*For radiologically occult tumor, the size was estimated at 2 mm, which is the inferior limit of voxel size in our MRI.

Bold denotes headings only: the functional subgroup headings (ACTH, PRL, GH) and the category subheadings (Demographics, Endocrine symptoms). No data value is bold.

NH, negative histology; PH, positive histology; ACTH, adrenocorticotropic hormone; PRL, prolactin; GH, growth hormone.

Pretreatment hormonal levels were compared between patients with NH and those with PH across the three subtypes of interest (**Figure 2**). Among patients with ACTH-secreting tumors, cortisoluria (median 170.2 [105.7–372.1] µg/24h vs. 137.5 [76.5–279.2] µg/24h, *p* = 0.33) and morning serum cortisol levels (median 599.0 [556.0–709.7] nmol/L vs. 509.5 [381.8–736.0] nmol/L, *p* = 0.06) were similar between those with NH and those with PH. Similarly, among patients with prolactinoma, those with NH displayed similar prolactin levels to their counterparts with PH (median 109.0 [55.9–196.5] ng/mL vs. 99.5 [65.0–171.0] ng/mL, *p* = 0.93). In contrast, baseline IGF-1 concentrations in patients with GH-secreting tumors were lower in NH patients than in those with PH tumors (median 350.4 [310.0–412.0] ng/mL vs. 852.5 [593.0–888.8] ng/mL, *p* < 0.01).

**Figure 2:**
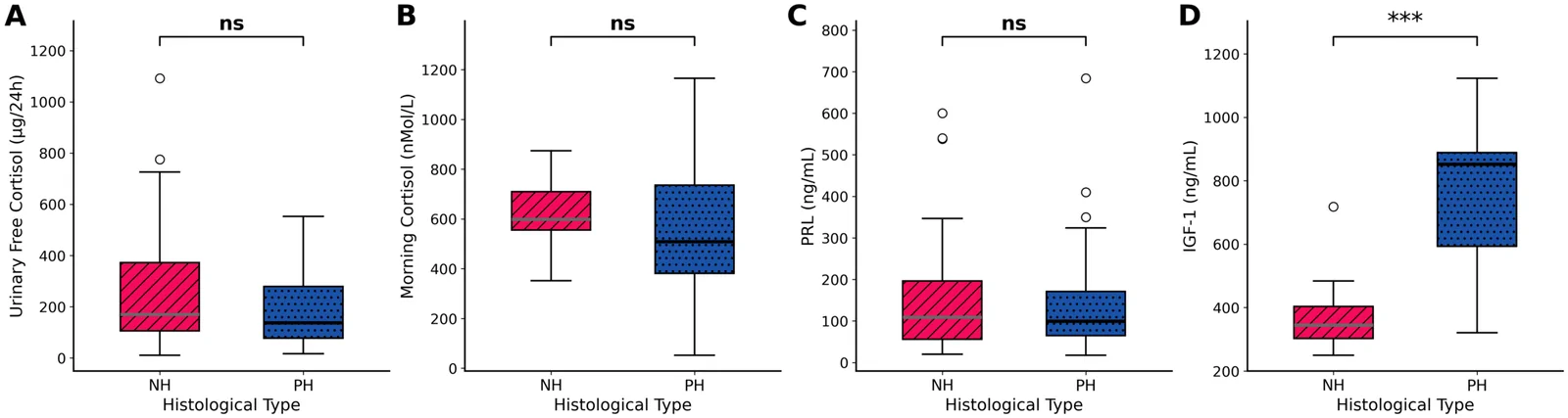
Preoperative hormonal levels according to histological findings in patients with microadenomas across the three most common functional subtypes (ACTH, PRL, and GH). **(A)** Urinary free cortisol in PH versus NH for ACTH-secreting tumors. **(B)** Morning serum cortisol levels in PH versus NH for ACTH-secreting tumors. **(C)** Prolactin levels in PH versus NH for PRL-secreting tumors. **(D)** IGF-1 levels in PH versus NH for GH-secreting tumors. NH, negative histology; PH, positive histology; ACTH, adrenocorticotropic hormone; PRL, prolactin; GH, growth hormone.

Likewise, the clinical presentation of patients did not significantly differ between NH and PH cases, including a similar rate of endocrine symptoms at diagnosis (92.1% vs. 95.5%, *p* = 0.49) and a similar duration of symptoms before surgery (4.1 years vs. 4.6 years, *p* = 0.76). Furthermore, the prevalence of incidentalomas, defined as pituitary tumors discovered in the absence of symptoms, was also comparable (7.9% vs. 6.7%, *p* = 0.76).

In accordance with our previous findings, the maximal diameter of the tumor was significantly smaller in NH compared to PH in the three subgroups.

### Postoperative outcomes

Absolute remission rates at 6 weeks (**Figure 3A**) were compared between NH and PH in the cohort restricted to patients with microadenomas.

**Figure 3:**
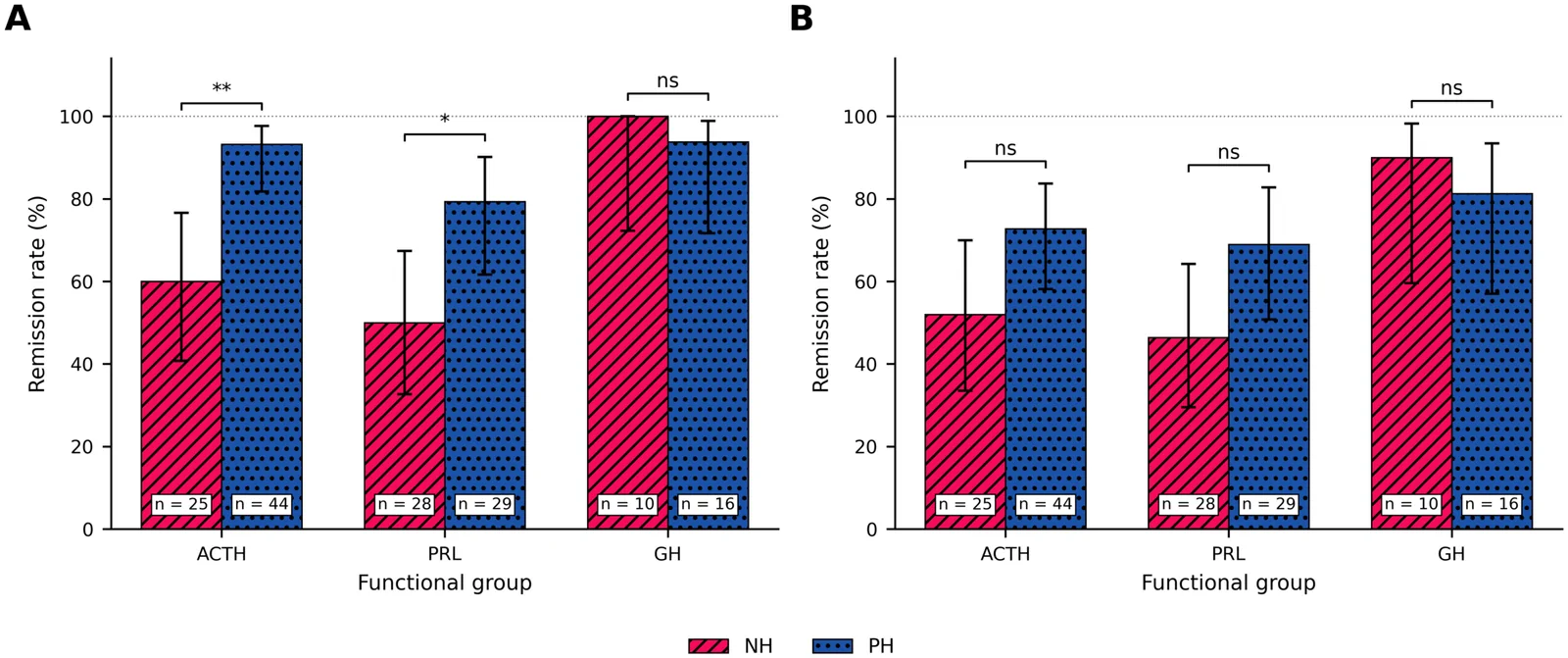
Remission rates according to histological findings in patients with microadenomas in the three most common functional subtypes. **(A)** Early postoperative remission rates at 6 weeks after surgery. **(B)** Remission rates at the last follow-up visit. The median follow-up duration was 4.0 years for ACTH, 3.9 years for PRL and 3.6 years for GH. The number n represents the total number of patients (100%) in each subgroup. NH, negative histology; PH, positive histology; ACTH, adrenocorticotropic hormone; PRL, prolactin; GH, growth hormone.

In ACTH-secreting tumors, remission at 6 weeks was achieved in 60% of patients with NH compared with 93.2% of patients with PH (*p* < 0.01). In prolactin-secreting tumors, remission rates were 50% in the NH group and 79.3% in the PH group (*p* = 0.03). By contrast, remission rates were comparable between the two groups in GH-secreting tumors (100% vs. 93.8%, *p* = 1.00).

Remission rates were also assessed at the last follow-up visit (**Figure 3B**). The median follow-up duration was 4.0 years (IQR 2.0–11.0) for ACTH, 3.9 years (IQR 0.7–9.9) for PRL, 3.6 years (IQR 1.6–6.6) for GH. Long term remission rates tended to be lower in NH tumors compared to PH tumors for both ACTH-secreting adenomas (52% vs. 72.7%, *p* = 0.07) and prolactinomas (46.4% vs. 69.0%, *p* = 0.11). These differences corresponded to substantial absolute reductions in remission rates (−20% and −22%, respectively), although they did not reach statistical significance at this time point. By contrast, in GH-secreting tumors, remission rates at the last follow-up visit were comparable between NH and PH cases (90.0% vs. 81.2%, *p* = 1.0).

No significant differences in follow-up duration were observed between NH and PH tumors within each subtype (see **Table 2**).

### Causes of surgical failure

The causes of long-term unfavorable surgical outcomes differed markedly across functional subtypes (**Figure 4A**). In ACTH-secreting tumors, suspected tumor mislocalization accounted for the majority of surgical failures (58.3%, 7/12 patients) while only one case (8.3%) of tumor mislocalization was confirmed with histology (in a case of an ectopic PitNET in the sphenoid sinus). Residual tumor and recurrence accounted for 16.7% (2/12) and 8.3% (1/12) of unfavorable outcomes, respectively, while the underlying mechanism remained unclear in one case (8.3%). Among these 12 patients, IPSS had been performed in 6/8 (75.0%) patients with a suspected or confirmed surgical error versus 2/4 (50.0%) patients with residual disease, recurrence, or unknown cause (*p* = 0.55). Lateralization on IPSS was achieved in 5/8 (62.5%) and 2/4 (50.0%) patients, respectively (*p* = 1.00).

**Figure 4:**
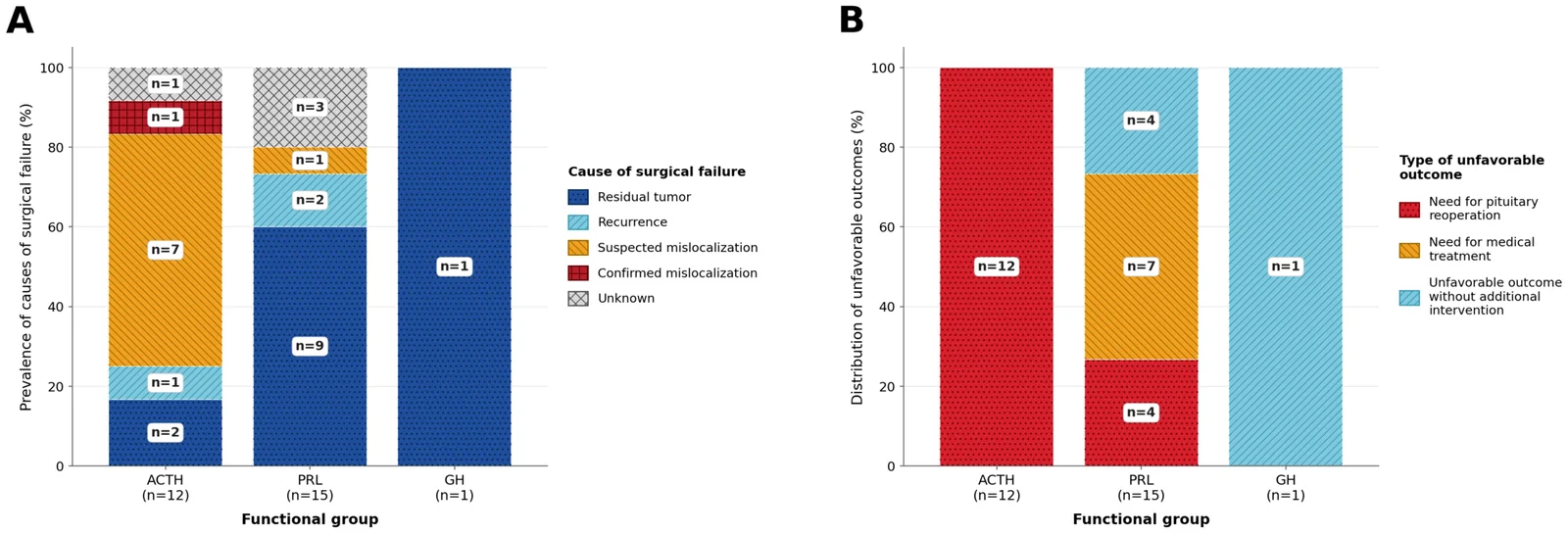
Causes of surgical failure and management of persistent disease in microadenomas of the three most common functional subtypes. **(A)** Prevalence of identified causes of surgical failure (residual tumor, recurrence, suspected or confirmed mislocalization, and unknown etiology). **(B)** Distribution of unfavorable outcomes (need for pituitary reoperation, need for medical treatment, or unfavorable outcome without additional intervention). ACTH, adrenocorticotropic hormone; PRL, prolactin; GH, growth hormone; PitNET, pituitary neuroendocrine tumor.

In prolactinomas, most unfavorable outcomes were related to residual tumor (9/15) and recurrence (2/15). One case (6.7%) was attributed to suspected tumor mislocalization and three cases had no obvious explanation. In GH-secreting tumors, the only unfavorable outcome was related to residual tumor.

### Management of persistent or recurrent disease

Second-line treatments for patients with NH and persistent disease are summarized in **Figure 4B**. Repeat surgery was required in all ACTH-secreting cases (12/12) and in 27% (4/15) of PRL-secreting cases. The remaining PRL cases were either managed medically (7/15) or by surveillance alone (4/15). The only patient with persistent GH-secreting disease was managed medically.

### Reoperations

Following an initial surgery with NH, 16 patients underwent reoperation and showed variable remission outcomes according to functional subtype and histological findings at subsequent surgery.

In ACTH-secreting tumors (n = 12), remission was achieved in 4 of 11 patients with persistent NH and in the only patient with histologically confirmed tumor at second surgery, giving a total remission rate of 41.7% (5/12). The second procedure consisted of nine repeat targeted adenomectomies (4/9 in remission, including the PH case), with two hypophysectomies (1/2 in remission) and one hemi-hypophysectomy (0/1 in remission). A third surgery was subsequently performed in three patients who had persistent disease after a second adenomectomy. It consisted of two repeat adenomectomies (one PH in remission, one NH without remission) and one hypophysectomy (NH, no remission).

In PRL-secreting tumors (n = 4), all patients underwent repeat selective adenomectomy. Remission was achieved in 1 of 1 patient with NH and in 2 of 3 patients with histologically confirmed tumors. No GH-secreting tumors underwent reoperation.

### Postoperative hormonal markers

In patients with Cushing’s disease and PH, postoperative morning serum cortisol levels displayed a consistent decline over time irrespective of long-term remission status (**Figure 5A**). Although cortisol levels tended to be higher in the patients with unfavorable outcomes, the difference between remission and unfavorable groups did not reach statistical significance at early postoperative time points (day 1: *p* = 0.10; day 5: *p* = 0.18), although a modest difference emerged at 6 weeks (*p* = 0.03).

**Figure 5:**
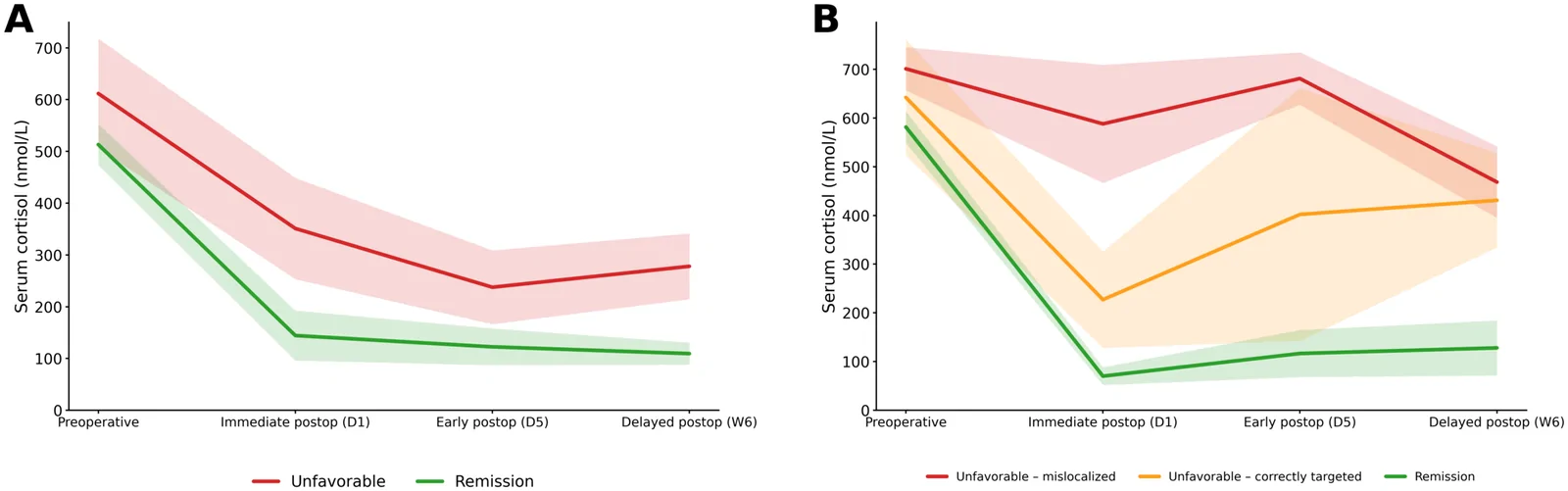
Early postoperative morning serum cortisol levels according to histological findings and clinical outcome. **(A)** Early postoperative morning serum cortisol in patients with PH, stratified by clinical outcome (remission vs. unfavorable). **(B)** Early postoperative morning serum cortisol in patients with NH, stratified into remission and unfavorable groups; the unfavorable group is subdivided into correct targeting (persistent disease despite exploration of the suspected tumor site) and mislocalization (postoperative evaluation suggesting the initial exploration targeted an incorrect anatomical location). NH, negative histology; PH, positive histology; Preop, preoperative (and pre-treatment); D1, postoperative day 1; D5, postoperative day 5; W6, postoperative week 6.

Among patients with ACTH-secreting tumors and NH results, cortisol dynamics after the first surgery differed significantly across groups at all postoperative time points (all *p* < 0.01; **Figure 5B**). Although mean cortisol levels tended to be higher in patients with mislocalized tumors compared with correctly targeted tumors, this difference did not reach statistical significance at any time point (day 1: *p* = 0.08; day 5: *p* = 0.47; week 6: *p* = 0.77). The effect size for this comparison was large at day 1 (*g* = 1.21), suggesting that the lack of significance may reflect limited statistical power.

### Adjusted outcome analyses

In the overall cohort of ACTH-, PRL-, and GH-secreting tumors, multivariable logistic regression analyses adjusting for maximum tumor diameter were performed to evaluate the association between histological findings and remission rates (**Table 3**).

**Table 3:** Multivariable logistic regression analyses of remission according to histological findings after adjustment for maximum tumor diameter.

Functional subgroup	Adjusted OR (PH vs NH)	95% CI	*p*
Short-term remission (6 weeks)			
**ACTH** (n = 96)	5.73	1.70–19.35	< 0.01
**PRL** (n = 114)	3.24	1.20–8.75	0.02
**GH** (n = 105)	—	—	—
Long-term remission (last follow-up)			
**ACTH** (n = 96)	2.60	0.95–7.07	0.06
**PRL** (n = 114)	1.63	0.64–4.17	0.31
**GH** (n = 105)	0.56	0.10–3.05	0.50

The model could not be reliably estimated for GH tumors at 6 weeks because all NH patients achieved remission (complete separation).

ACTH, adrenocorticotropic hormone; PRL, prolactin; GH, growth hormone; OR, odds ratio.

At 6 weeks postoperatively, PH remained independently associated with higher remission rates in ACTH-secreting tumors (OR 5.73, 95% CI 1.70–19.35, *p* < 0.01). Similarly, in prolactin-secreting tumors, PH was independently associated with early remission after adjustment for tumor size (OR 3.24, 95% CI 1.20–8.75, *p* = 0.02). The model could not be reliably estimated for GH tumors at 6 weeks because all NH patients (10/10) achieved remission (complete separation).

Bold row labels indicate subheadings. Bold values indicate statistically significant associations (p < 0.05).

At the last follow-up visit, remission rates remained lower in NH compared with PH tumors in both ACTH- and PRL-secreting tumors after adjustment for tumor size, although these differences no longer reached statistical significance (ACTH: OR 2.60, 95% CI 0.95–7.07, *p* = 0.06; PRL: OR 1.63, 95% CI 0.64–4.17, *p* = 0.31). Likewise, no significant association was observed in GH-secreting tumors at long-term follow-up (OR 0.56, CI 0.10–3.05, *p* = 0.50).

Additional adjustment for age, sex, and BMI did not materially alter the overall findings (data not shown).

## Discussion

This large single-center retrospective study identifies NH as a frequent finding in patients operated for a pituitary neuroendocrine tumor (PitNET), especially in ACTH- (28.1%) and PRL-secreting tumors (28.1%), with a prevalence consistent with previous reports in these subtypes (see **Supplementary Table 1**). In addition, our results show that NH is not limited to corticotroph tumors and prolactinomas but also occurs for GH-secreting tumors (11.4%), plurihormonal tumors (7.1%), and even non-functioning tumors (3.3%). These subtypes have rarely been specifically studied in the literature, which has largely focused on Cushing’s disease.

Our results highlight a strong relationship between negative histology and microadenomas. On the one hand, the majority of NH cases (78%) were observed in microadenomas or radiologically occult lesions. On the other hand, the prevalence of NH across PitNET subtypes was strongly correlated (r = 0.87) with the proportion of microadenomas within each subtype, and within each of the three most common functional subtypes NH was significantly more frequent in microadenomas than in macroadenomas. Furthermore, even when analyses were restricted to microadenomas, the maximal diameter was still significantly smaller for NH in the three most common functional subtypes.

This convergent association is consistent with all three mechanisms discussed in the Introduction to explain NH findings. First, when a lesion is very small, the surgical specimen may inadvertently consist of adjacent normal pituitary tissue rather than tumor tissue, simply because the margin for error during sampling is reduced. Second, even when tumor tissue is correctly sampled, the limited amount of material and the more subtle architectural changes typical of very small lesions make histopathological identification more challenging. Third, microadenomas are inherently more difficult to localize on preoperative imaging, increasing the risk that surgical exploration targets an incorrect site and that normal pituitary tissue is resected instead of the actual lesion. This risk of tumor mislocalization is probably higher when complementary localization studies are negative or misleading, including IPSS lateralization (20–22) and equivocal PET findings (23, 24), although this was not evident from our data. These three mechanisms are not mutually exclusive and are all expected to become more relevant as tumor size decreases. Their respective contributions, however, likely vary across subtypes, as discussed further below.

Analysis of patient characteristics indicates that NH was not associated with a distinct clinical profile in our cohort. In particular, the proportion of patients presenting with endocrine symptoms at diagnosis, symptom duration, and the prevalence of incidentalomas were similar between the NH and PH groups. These findings suggest that NH does not reflect an earlier stage of tumor development (as previously suggested for hyperplasia (25)) but rather a diagnostic discordance independent of disease severity or duration. Preoperative hormone levels were also comparable between NH and PH groups for ACTH- and PRL-secreting tumors. However, in patients with acromegaly related to a GH-secreting tumor, IGF-1 levels were significantly lower in the NH group compared to the PH group. This difference suggests that low-GH-secreting microadenomas are more likely to be missed at histology, either because of a smaller size or because of a weaker hormonal expression.

Surgical outcomes were particularly unfavorable in ACTH-secreting tumors with NH, with significantly lower remission rates at 6 weeks (60% vs. 93.2%, *p* < 0.01) and a systematic need for reoperation. Although the prognostic value of NH in these tumors remains controversial, with some studies reporting outcomes comparable to PH (14–19, 26, 27), our results are consistent with reports suggesting worse outcomes (13).

Our findings suggest that the lower remission rates observed in ACTH-secreting NH tumors may be largely attributable to tumor mislocalization. Indeed, when the causes of unfavorable outcomes are examined in detail, suspected or confirmed radiological mislocalization accounted for 66.7% of surgical failures in NH cases (8/12), whereas 25% of the remaining failures were related to residual tumor or recurrences (3/12) and one case had an unknown cause (1/12). Once mislocalized cases are set aside, the residual failure rate in NH cases (4/25, 16%) is no longer higher than that observed in PH cases (12/44, 27%), in whom mislocalization is by definition absent. In other words, once mislocalization is set aside, the residual mechanisms of failure and their frequency appear broadly similar between NH and PH cases. This descriptive observation suggests that the excess of unfavorable outcomes in NH cases may largely be explained by mislocalization, and that, in its absence, NH per se may not carry an intrinsically worse prognosis. This interpretation should nonetheless be made with caution, as mislocalization was in part identified retrospectively among unfavorable cases, which may partly account for its apparent contribution.

Only four case series have specifically studied the prognosis of NH patients after repeat surgery in ACTH-secreting tumors, totaling 13 patients (see **Supplementary Table 1** for the details). The uncontrolled pooled remission rate of these very heterogeneous series is 69.2% (9/13), compared with 41.7% (5/12) at the second surgery in this study. Furthermore, in our cohort, remission was achieved in the two patients in whom adenomatous tissue was eventually identified at the second or third surgery, whereas it was achieved in less than half of those with persistent NH despite repeated procedures. This pattern suggests that persistent tumor mislocalization may account for the failure of repeat surgery in a substantial subset of NH cases. Consistently, NH tended to persist across repeated procedures, with 11 of 12 patients (92%) displaying persistent NH at the second surgery and 2 of 3 patients still displaying NH at the third surgery. Such persistence is more readily explained by tumor mislocalization than by sampling or histopathological limitations, which would be expected to be at least partially overcome through repeated and increasingly extensive surgical exploration.

Postoperative morning serum cortisol levels may also provide complementary information for the early identification of surgical failure in ACTH-secreting PitNETs (**Figure 5**). In PH patients, these levels typically decline rapidly, reflecting at least partial tumor removal (19, 28). In NH patients, higher early postoperative cortisol levels were observed in patients with unfavorable outcomes, and a non-significant trend toward higher values was noted in the mislocalized subgroup compared with correctly targeted cases (*p* = 0.08 at day 1). Whether early postoperative cortisol dynamics can reliably help distinguish mislocalized from correctly targeted cases in NH patients remains a hypothesis that warrants validation in larger prospective cohorts.

In prolactinomas, outcomes were also less favorable in NH cases, with only 46.4% of remission at long term in the NH group compared with 69.0% in the PH group (*p* = 0.03). The prognostic impact of NH in prolactinomas was only investigated in two short case series which reported remission rates of 55.6% (4) and 57.1% (5), respectively, slightly more favorable than in our cohort. A parallel pattern to that observed in ACTH-secreting tumors, although less clear-cut, may also be present regarding tumor mislocalization: only one case of suspected radiological mislocalization was identified (6.7% of unfavorable outcomes), but three additional unfavorable cases (20.0%) had no clear explanation, and whether some of these unexplained failures could also reflect unrecognized mislocalization remains speculative. Notably, the remission rate at second surgery was 75% and did not seem correlated with the histopathological identification of a tumor, although the sample size (4 patients) is too small to draw firm conclusions.

By contrast, surgical outcomes were generally favorable in GH-secreting tumors in our cohort, with comparable remission rates between NH and PH cases (90.0% vs. 81.2% at long term, *p* = 1.0). Available data in the literature are scarce, with only one study reporting a remission rate of 50% (2/4 patients) in patients with “inconclusive” histology (7), markedly lower than the 90% (9/10) observed in our cohort at the last follow-up.

## Clinical implications

From a practical perspective, our findings suggest that the clinical implications of NH differ substantially according to PitNET subtype and should therefore not be interpreted uniformly.

In ACTH-secreting tumors, NH should raise strong suspicion of inaccurate tumor localization, particularly in patients with persistent hypercortisolism after surgery, as this mechanism accounted for the majority of unfavorable outcomes in our cohort. Reoperation outcomes in our cohort were modest overall (5/12, 41.7% remission), with only 4 of the 11 patients in whom no tumor could be identified at the second surgery achieving remission, whereas the single patient in whom a tumor was eventually found also achieved remission. Therefore, in cases of NH findings at the first surgery, careful postoperative reassessment of tumor localization, ideally combining IPSS reinterpretation and 11C-methionine PET imaging appears essential before considering repeat surgery.

In PRL-secreting tumors, remission rates were significantly lower in NH cases at 6 weeks but became comparable to those of PH cases at last follow-up. Persistent disease was most often controlled with medical therapy (only 27% required repeat surgery), and reoperation, when performed, remained effective (75% remission in our cohort). This less unfavorable profile likely reflects the lower prevalence of tumor mislocalization in this subtype. Nevertheless, mislocalization should still be considered in PRL-secreting NH cases with unfavorable outcomes, particularly when no residual or recurrent tumor is identified on the follow-up MRIs.

In GH-secreting tumors, surgical outcomes remained favorable despite NH findings, with the only case of persistent disease adequately controlled medically. Although these observations are based on a limited number of cases, they are consistent with the hypothesis that NH in this subtype predominantly reflects technical and histopathological limitations, rather than incorrect surgical targeting.

## Limitations

This study has several limitations. First, this was a single-center study, which may limit the generalizability of our findings, and its retrospective design may have introduced selection and information biases, although exclusion because of loss to follow-up was limited (three patients in the three subtypes of interest: one ACTH and two PRL cases). By far, the most common reasons for exclusion were that the procedure was not a primary operation or that no pathological report was available.

Second, the study period spans more than two decades (1996–2023), during which imaging modalities, histopathological evaluation (notably with the introduction of transcription factors) and endocrine assays have evolved. However, this long inclusion period was necessary to obtain a sufficiently large cohort and adequate follow-up duration.

Third, all patients in this study were treated using a consistent microscopic endonasal transsphenoidal approach, which limits intra-cohort variability related to surgical technique but also limits the generalizability of our findings to centers using a primarily endoscopic approach. Whether the prevalence and the relative contribution of the proposed mechanisms of NH differ between microscopic and endoscopic transsphenoidal surgery remains incompletely characterized, although the few endoscopic series have reported broadly similar NH prevalence (9, 18, 29).

## Conclusion

This study shows that NH after transsphenoidal surgery for PitNET is a common finding, observed in 12.6% of our cohort overall and in close to 30% of ACTH- and PRL-secreting tumors. NH is strongly associated with microadenomas and extends beyond ACTH- and PRL-secreting tumors to also occur, although less frequently, in GH-secreting, plurihormonal, and non-functioning tumors.

Beyond its prevalence, our findings argue against considering NH as a uniform entity. Rather, NH appears to reflect a heterogeneous phenomenon resulting from three different mechanisms: inadvertent sampling of normal pituitary tissue, histopathological limitations and tumor mislocalization. In ACTH-secreting tumors, inaccurate tumor localization appears to be the dominant mechanism and accounts for most of the excess of unfavorable outcomes observed in NH cases. In PRL-secreting tumors, mislocalization is uncommon but may contribute to the lower early remission rates observed in NH cases. In GH-secreting tumors, NH is most likely to reflect technical and histopathological limitations of identifying very small lesions.

Taken together, these findings support a subtype-specific interpretation of NH and provide a practical framework for postoperative decision-making. Future multicenter studies incorporating systematic transcription factor immunostaining and advanced functional imaging such as 11C-methionine PET may help further refine the mechanisms underlying NH and validate the subtype-specific management strategies proposed here.

## Data Availability

The raw data supporting the conclusions of this article will be made available by the authors, without undue reservation.
