# Supplementary material for: Prevalence and clinical relevance of negative histology after transsphenoidal surgery for pituitary neuroendocrine tumors
Source: Front Endocrinol (Lausanne). 2026 Sep 15;17:1933467. doi: 10.3389/fendo.2026.1933467 (PMC13619420; doi:10.3389/fendo.2026.1933467)
Supplement: Supplementary file 1 [file Table1.docx]

| **ACTH** | **NH prevalence**  n/N (%) | **Remission rate in NH**  n/N (%) | **Remission rate in reoperated NH**  n/N (%) | **NH prevalence in reoperated NH**  n/N (%) |
| --- | --- | --- | --- | --- |
| Bigos (1980)^30^ | 3/24 (12.5) | — | — | — |
| Hardy (1982)^31^ | 15/75 (20.0) | — | — | — |
| Salassa (1983)^32^ | 10/98 (10.2) | — | — | — |
| Boggan (1983)^33^ | 18/100 (18.0) | — | — | — |
| Kageyama (1984)^34^ | 11/90 (12.2) | — | — | — |
| Fahlbusch (1986)^35^ | 8/96 (8.3) | — | — | — |
| Chandler (1987)^36^ | 9/33 (27.3) | — | — | — |
| Guilhaume (1988)^37^ | 22/58 (37.9) | — | — | — |
| Mampalam (1988)^14^ | 38/216 (17.6) | 26/38 (68.4) | — | — |
| Burke et al. (1990)^15^ | 17/64 (26.6) | 14/17 (82.4) | — | — |
| Lindholm (1992)^38^ | 20/45 (44.4) | — | — | — |
| Comtois et al. (1993)^16^ | 13/60 (21.7) | — | — | — |
| Bakiri et al. (1996)^39^ | 10/50 (20.0) | 3/10 (30.0) | — | — |
| Gsponer et al. (1999)^6^ | 1/16 (6.2) | — | — | — |
| Netea-Maier et al. (2006)^29^ | 10/35 (28.6) | 5/10 (50) | 2/4 (50.0) | NR |
| Acebes et al. (2007)^40^ | 9/44 (20.5) | 5/9 (55.6) | — | — |
| Pouratian et al. (2007)^8^ | 111/490 (22.7) | 56/111 (50.5) | — | — |
| Prevedello et al. (2008)^41^ | 31/167 (18.6) | 20/31 (64.5) | 1/1 (100.0) | 1/1 (100.0) |
| Hofmann et al. (2008)^42^ | 58/426 (13.6) | — | — | — |
| Patil et al. (2008)^26^ | 12/36 (33.3) | — | — | — |
| Patil et al. (2008)^43^ | 30/215 (14.0) | — | — | — |
| Jagannathan et al. (2009)^44^ | 13/261 (5.0) | 7/13 (53.8) | 6/6 (100.0) | 4/6 (66.7) |
| Bou Khalil et al. (2011)^45^ | 31/127 (24.4) | 14/31 (45.2) | — | — |
| Yamada et al. (2012)^46^ | 11/124 (8.9) | 1/11 (9.1) | — | — |
| Hassan-Smith et al. (2012)^47^ | NR | NR (60) | — | — |
| Witek et al. (2012)^48^ | 9/36 (25.0) | 3/9 (33.3) | — | — |
| Ciric et al. (2012)^27^ | 11/105 (10.5) | 9/11 (81.8) | — | — |
| Kuo et al. (2015)^9^ | 6/40 (15.0) | 3/6 (50.0) | — | — |
| Sarkar et al. (2016)^18^ | 7/64 (10.9) | 5/7 (71.4) | 0/2 (0.0) | 1/2 (50.0) |
| Brichard et al. (2018)^19^ | 19/58 (33.8) | 14/19 (73.7) | — | — |
| Serban et al. (2020)^49^ | 9/65 (13.8) | 5/9 (55.6) | — | — |
| Dai et al. (2022)^50^ | 161/1106 (14.6) | 80/161 (49.7) | — | — |
| Little et al. (2024)^51^ | 74/496 (14.9) | — | — | — |
| Lamback et al. (2025)^52^ | 14/84 (16.7) | — | — | — |
| Ghidoni et al. (2026)^53^ | 3/22 (13.6) | — | — | — |
| **PRL** | **NH prevalence**  n/N (%) | **Remission rate in NH**  n/N (%) | **Remission rate in reoperated NH**  n/N (%) | **NH prevalence in reoperated NH**  n/N (%) |
| Turner et al. (1999)^4^ | 9/24 (37.5) | 5/9 (55.6) | — | — |
| Gsponer et al. (1999)^6^ | 3/111 (2.7) | — | — | — |
| Losa et al. (2002)^5^ | 7/120 (5.8) | 4/7 (57.1) | — | — |
| Van Trigt et al. (2025)^54^ | 14/100 (14.0)* | — | — | — |
| **GH** | **NH prevalence**  n/N (%) | **Remission rate in NH**  n/N (%) | **Remission rate in reoperated NH**  n/N (%) | **NH prevalence in reoperated NH**  n/N (%) |
| Gsponer et al. (1999)^6^ | 1/31 (3.2) | — | — | — |
| Kreutzer et al. (2001)^7^ | 4/57 (7.0)** | 2/4 (50.0) | — | — |

**Table S1. Narrative literature review about negative histology in ACTH-, PRL- and GH-secreting adenomas.**

*Broad NH definition including unsatisfactory material, hypophysitis, non-PIT1 PitNET, hyperplasia.

**The authors use the term "inconclusive histology", which is not clearly defined in their work.

**Abbreviations:** ACTH, adrenocorticotropic hormone; PRL, prolactin; GH, growth hormone;

**References**

*References 30 to 54 are cited only in this Supplementary Material. Numbering follows the reference list of the main article. The remaining citations in Table S1 (4–9, 14–16, 18, 19, 26, 27, 29) are listed in the reference list of the main article.*

30. Bigos ST, Somma M, Rasio E, Eastman RC, Lanthier A, Johnston HH, et al. Cushing's disease: management by transsphenoidal pituitary microsurgery. *J Clin Endocrinol Metab*. (1980) 50:348–54. doi: 10.1210/jcem-50-2-348

31. Hardy J. Cushing's disease: 50 years later. *Can J Neurol Sci*. (1982) 9:375–80. doi: 10.1017/S0317167100044279

32. Salassa RM, Laws ER, Carpenter PC, Northcutt RC. Cushing's disease – 50 years later. *Trans Am Clin Climatol Assoc*. (1983) 94:122.

33. Boggan JE, Tyrrell JB, Wilson CB. Transsphenoidal microsurgical management of Cushing's disease: report of 100 cases. *J Neurosurg*. (1983) 59:195–200. doi: 10.3171/jns.1983.59.2.0195

34. Kageyama N, Kuwayama A, Takahashi T, Ichihara K, Kato T, Yokoe T. Microsurgical results in 90 cases of Cushing's disease. In: *Trends in Diagnosis and Treatment of Pituitary Adenomas*. Amsterdam: Free University Press (1984). p. 325–38.

35. Fahlbusch R, Buchfelder M, Müller OA. Transsphenoidal surgery for Cushing's disease. *J R Soc Med*. (1986) 79:262–9. doi: 10.1177/014107688607900504

36. Chandler WF, Schteingart DE, Lloyd RV, McKeever PE, Ibarra-Perez G. Surgical treatment of Cushing's disease. *J Neurosurg*. (1987) 66:204–12. doi: 10.3171/jns.1987.66.2.0204

37. Guilhaume B, Bertagna X, Thomsen M, Bricaire C, Vila-Porcile E, Olivier L, et al. Transsphenoidal pituitary surgery for the treatment of Cushing's disease: results in 64 patients and long term follow-up studies. *J Clin Endocrinol Metab*. (1988) 66:1056–64. doi: 10.1210/jcem-66-5-1056

38. Lindholm J. Endocrine function in patients with Cushing's disease before and after treatment. *Clin Endocrinol*. (1992) 36:151–9. doi: 10.1111/j.1365-2265.1992.tb00950.x

39. Bakiri F, Tatai S, Aouali R, Semrouni M, Derome P, Chitour F, et al. Treatment of Cushing's disease by transsphenoidal, pituitary microsurgery: prognosis factors and long-term follow-up. *J Endocrinol Invest*. (1996) 19:572–80. doi: 10.1007/BF03349020

40. Acebes JJ, Martino J, Masuet C, Montanya E, Soler J. Early post-operative ACTH and cortisol as predictors of remission in Cushing's disease. *Acta Neurochir (Wien)*. (2007) 149:471–9. doi: 10.1007/s00701-007-1133-1

41. Prevedello DM, Pouratian N, Sherman J, Jane JA, Vance ML, Lopes MB, et al. Management of Cushing's disease: outcome in patients with microadenoma detected on pituitary magnetic resonance imaging. *J Neurosurg*. (2008) 109:751–9. doi: 10.3171/JNS/2008/109/10/0751

42. Hofmann BM, Hlavac M, Martinez R, Buchfelder M, Müller OA, Fahlbusch R. Long-term results after microsurgery for Cushing disease: experience with 426 primary operations over 35 years. *J Neurosurg*. (2008) 108:9–18. doi: 10.3171/jns/2008/108/01/0009

43. Patil CG, Prevedello DM, Lad SP, Vance ML, Thorner MO, Katznelson L, et al. Late recurrences of Cushing's disease after initial successful transsphenoidal surgery. *J Clin Endocrinol Metab*. (2008) 93:358–62. doi: 10.1210/jc.2007-2013

44. Jagannathan J, Smith R, DeVroom HL, Vortmeyer AO, Stratakis CA, Nieman LK, et al. Outcome of using the histological pseudocapsule as a surgical capsule in Cushing disease. *J Neurosurg*. (2009) 111:531–9. doi: 10.3171/2008.8.JNS08339

45. Bou Khalil R, Baudry C, Guignat L, Carrasco C, Guibourdenche J, Gaillard S, et al. Sequential hormonal changes in 21 patients with recurrent Cushing's disease after successful pituitary surgery. *Eur J Endocrinol*. (2011) 165:729–37. doi: 10.1530/eje-11-0424

46. Yamada S, Fukuhara N, Nishioka H, Takeshita A, Inoshita N, Ito J, et al. Surgical management and outcomes in patients with Cushing disease with negative pituitary magnetic resonance imaging. *World Neurosurg*. (2012) 77:525–32. doi: 10.1016/j.wneu.2011.06.033

47. Hassan-Smith ZK, Sherlock M, Reulen RC, Arlt W, Ayuk J, Toogood AA, et al. Outcome of Cushing's disease following transsphenoidal surgery in a single center over 20 years. *J Clin Endocrinol Metab*. (2012) 97:1194–201. doi: 10.1210/jc.2011-2957

48. Witek P, Zieliński G, Maksymowicz M, Zgliczyński W. The relationship between efficacy of surgical treatment of Cushing disease and pathological–immunohistochemical and ultrastructural–confirmation of corticotroph tumour presence. *Neurol Neurochir Pol*. (2012) 46:37–46. doi: 10.5114/ninp.2012.27177

49. Serban AL, Del Sindaco G, Sala E, Carosi G, Indirli R, Rodari G, et al. Determinants of outcome of transsphenoidal surgery for Cushing disease in a single-centre series. *J Endocrinol Invest*. (2020) 43:631–9. doi: 10.1007/s40618-019-01151-1

50. Dai C, Feng M, Sun B, Bao X, Yao Y, Deng K, et al. Surgical outcome of transsphenoidal surgery in Cushing's disease: a case series of 1106 patients from a single center over 30 years. *Endocrine*. (2022) 75:219–27. doi: 10.1007/s12020-021-02848-1

51. Little AS, Karsy M, Evans JJ, Kim W, Pacione DR, Kim AH, et al. Multicenter registry of adenomas of the pituitary and related disorders: initial description of Cushing disease cohort, surgical outcomes, and surgeon characteristics. *Neurosurgery*. (2024) 95:372. doi: 10.1227/neu.0000000000002888

52. Lamback E, Kasuki L, Miranda RL, de Figueiredo CS, Wildemberg LE, Gadelha MR. The prevalence and predictors of Cushing disease recurrence: a 10-year experience of a pituitary tumor center of excellence. *Endocrine*. (2025) 89:283–90. doi: 10.1007/s12020-025-04234-7

53. Ghidoni F, Madoglio A, Chiloiro S, Di Bergamo LT, Vezzoli M, Serioli S, et al. Cushing's disease and negative MRI: a single-center series, systematic literature review, and meta-analysis. *Neurosurg Rev*. (2026) 49:246. doi: 10.1007/s10143-026-04168-2

54. van Trigt VR, Bakker LEH, Pelsma ICM, Zandbergen IM, Jentus MM, Kruit MC, et al. The changing treatment paradigm for prolactinoma—a prospective series of 100 consecutive neurosurgical cases. *J Clin Endocrinol Metab*. (2025) 110:e1833–44. doi: 10.1210/clinem/dgae652
